# Liver fat and clinical outcomes in individuals with stage I-III colon or rectal cancer

**DOI:** 10.1093/jnci/djaf324

**Published:** 2025-11-10

**Authors:** Deborah Ophoff, Daniel Bos, Tjarda van Heek, Ben J Witteman, Johannes H W de Wilt, Karteek Popuri, Mirza Faisal Beg, Renate M Winkels, Fränzel J B van Duijnhoven, Edward L Giovannucci, Ellen Kampman, Dieuwertje E Kok

**Affiliations:** Division of Human Nutrition and Health, Wageningen University & Research, Wageningen, The Netherlands; Department of Epidemiology, Erasmus MC University Medical Center, Rotterdam, The Netherlands; Department of Radiology and Nuclear Medicine, Erasmus MC University Medical Center, Rotterdam, The Netherlands; Division of Human Nutrition and Health, Wageningen University & Research, Wageningen, The Netherlands; Division of Human Nutrition and Health, Wageningen University & Research, Wageningen, The Netherlands; Department of Gastroenterology and Hepatology, Hospital Gelderse Vallei, Ede, The Netherlands; Department of Surgery, Radboud University Medical Center, Nijmegen, The Netherlands; Department of Computer Science, Memorial University of Newfoundland, St. John’s, NL, Canada; School of Engineering, Simon Fraser University, Burnaby, BC, Canada; Division of Human Nutrition and Health, Wageningen University & Research, Wageningen, The Netherlands; Division of Human Nutrition and Health, Wageningen University & Research, Wageningen, The Netherlands; Department of Nutrition, Harvard T.H. Chan School of Public Health, Boston, MA, United States; Department of Epidemiology, Harvard T.H. Chan School of Public Health, Boston, MA, United States; Division of Human Nutrition and Health, Wageningen University & Research, Wageningen, The Netherlands; Division of Human Nutrition and Health, Wageningen University & Research, Wageningen, The Netherlands

## Abstract

**Background:**

Liver fat accumulation has been associated with impaired colorectal cancer prognosis. Associations may differ for colon and rectal cancer due to different disease mechanisms and dissemination patterns. Here, we investigated associations between liver fat and cancer recurrence, recurrence-free survival (RFS), and overall survival (OS) among 1596 individuals with stage I-III colon or rectal cancer.

**Methods:**

Within a prospective cohort study, we used data from adults recently diagnosed with colon (*n* = 1080) or rectal (*n* = 516) cancer. Liver fat was evaluated using routine contrast-enhanced computed tomography (CT)-scans taken at diagnosis. Cox proportional hazards regression analyses adjusted for clinical and lifestyle-related variables were used to obtain hazard ratios (HRs) and 95% confidence intervals (95% CIs).

**Results:**

During a median follow-up of 6.4 and 8.8 years, 247 (15%) recurrences (12% for colon and 22% for rectal cancer) and 418 (26%) deaths (25% for colon and 29% for rectal cancer) occurred, respectively. More liver fat was associated with an increased recurrence risk (HR_T3vsT1_ = 1.60, 95% CI = 1.02 to 2.50), worse RFS (HR_T3vsT1_ = 1.45, 95% CI = 1.05 to 2.00), and OS (HR_T3vsT1_ = 1.67, 95% CI = 1.20 to 2.33) among individuals with colon cancer. Liver fat was not associated with recurrence (HR_T3vsT1_ = 0.70, 95% CI = 0.42 to 1.18), RFS (HR_T3vsT1_ = 0.87, 95% CI = 0.59 to 1.30), or OS (HR_T3vsT1_ = 1.15, 95% CI = 0.74 to 1.80) among individuals with rectal cancer.

**Conclusion:**

More liver fat was associated with poor clinical outcomes in patients with stage I-III colon cancer. Further studies are needed to confirm these findings and explore mechanistic routes linking liver fat to colon cancer prognosis.

## Introduction

Colorectal cancer (CRC) is the third most common cancer worldwide.^1^ Approximately 25%–50% of individuals with CRC will eventually develop locoregional recurrences or distant metastases^2^^,^^3^ depending on the primary tumor location and cancer stage.^4^ The liver is the most common site for CRC dissemination.^2^^,^^5^

Excessive liver fat accumulation, prevalent in ∼25% of the general population,^6^ has been proposed as determinant of colorectal liver metastases and survival,^7-12^ although findings are inconsistent.^10^^,^^13-16^ Inflammation and activation of stellate cells are hypothesized to enable formation of metastases in a fatty liver,^17^^,^^18^ consequently worsening CRC prognosis.^19^ Whereas literature on liver fat and recurrent liver metastases or survival in metastatic CRC is emerging,^9^^,^^11-14^^,^^20-23^ relatively few studies were conducted in non-metastatic CRC (sample sizes ranging from 202 to 3262 participants).^7^^,^^8^^,^^15^^,^^24-26^ Moreover, most of these studies did not consider lifestyle-related factors, such as alcohol intake and body mass index (BMI), that might confound associations between liver fat and clinical outcomes.^27-29^

Associations between liver fat and clinical outcomes of CRC might differ per tumor location, since vascular anatomy, underlying disease mechanisms, cancer dissemination patterns, treatment options, and prognosis differ between colon and rectal cancer.^4^^,^^5^^,^^30^ Hence, primary tumor location should be considered when investigating relationships of liver fat with clinical outcomes. So far, one study has investigated associations in 241 patients with rectal cancer specifically.^24^

Here, we investigated associations between liver fat and recurrence, recurrence-free survival (RFS), and overall survival (OS) in patients with stage I-III CRC, stratified by primary tumor location.

## Methods

### Design and population

For the current study, we used data from the COLON study. Study procedures have been described elsewhere.^31^ The COLON study is a prospective cohort study among 2107 adults newly diagnosed with stage I-IV CRC, recruited between 2010 and 2020 via 11 hospitals throughout the Netherlands. All participants provided written informed consent. The COLON study was approved by an institutional review board (region Arnhem-Nijmegen, 2009-349).

**Figure 1.**
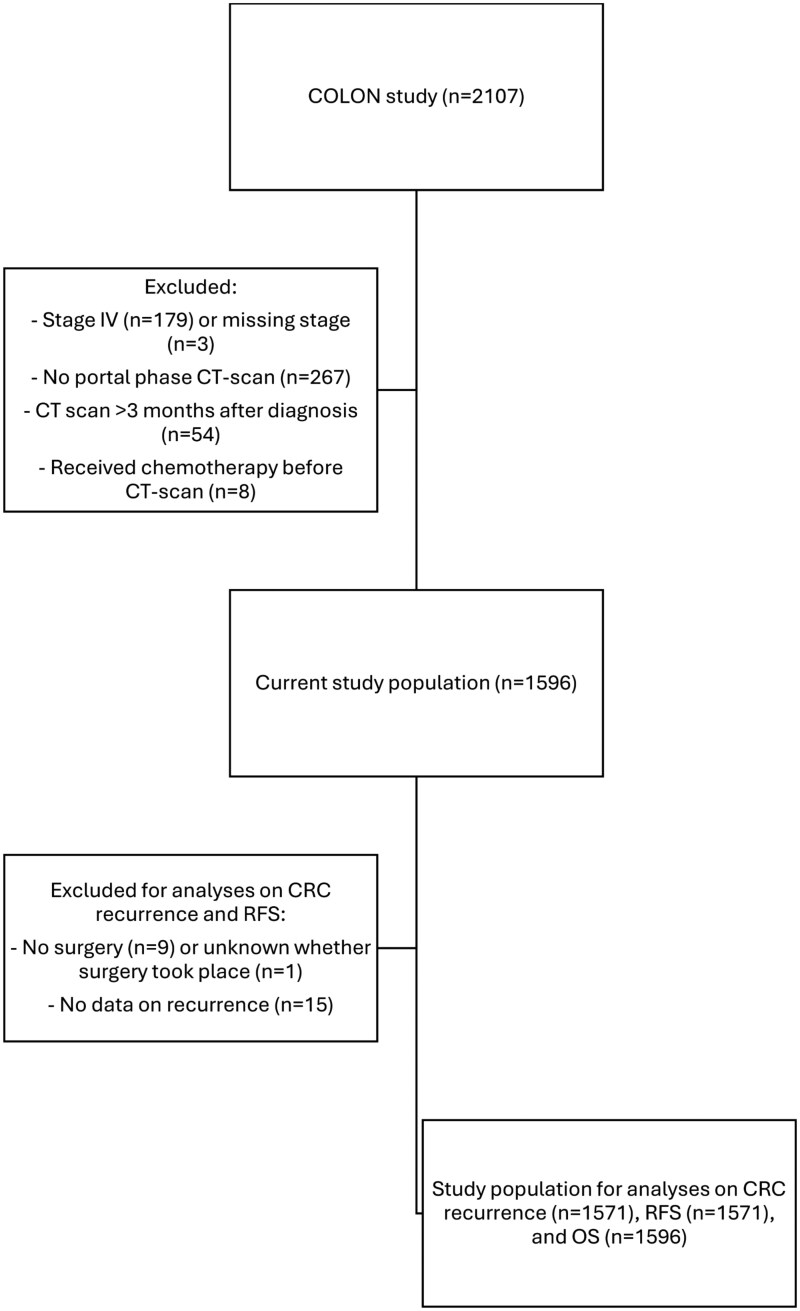
Flow chart showing the selection of participants with available data for the current study.

For this study, we excluded participants with stage IV (*n* = 179) or unknown (*n* = 3) cancer stage (Figure 1). We used contrast-enhanced (portal-phase) computed tomography (CT)-scans, routinely taken at diagnosis, to evaluate liver fat. Therefore, we also excluded participants without a portal-phase CT-scan at diagnosis (*n* = 267), who underwent a CT-scan >3 months after diagnosis (*n* = 54), or who received chemotherapy before the CT-scan date (*n* = 8), since chemotherapy may induce liver fat.^32^ Consequently, the current study population consisted of 1596 participants. For analyses focusing on recurrence and RFS, we additionally excluded participants who did not undergo surgery (*n* = 9), or had missing data on surgery (*n* = 1) or recurrence (*n* = 15), resulting in a study population of 1571.

### Assessment of liver fat

Liver fat, reflected by liver radiodensity on abdominal portal-phase CT-scans (in Hounsfield units, HU), was assessed using RadiAnt DICOM Viewer software.^33^ For 2 participants, abdominal scans were not available and liver radiodensity was measured on thorax portal-phase CT-scans. We placed a region of interest (ROI) of ∼2 cm^2^ in each of the 8 Couinaud segments,^34^ avoiding placement in bile ducts, vessels, or liver lesions. Radiodensity values within these 8 ROIs were averaged to calculate overall liver radiodensity per participant. A higher radiodensity value reflects less liver fat. All measurements were performed by one researcher (D.O.), trained by a radiology expert group (led by D.B.). To assess inter-observer variability, another independent non-radiologist observer evaluated a random subset (*n* = 50) of the scans (variation coefficient 2.4%).

As indicated, we were not able to retrieve scans for 267 participants because of logistic reasons, or because participants only had arterial-phase or non-enhanced CT-scans available (Figure 1). There were no remarkable differences between characteristics of participants with or without a portal-phase CT-scan available (Table S1).

### Endpoints

Data on CRC recurrence were obtained through linkage with the Netherlands Cancer Registry (NCR), via the Netherlands Comprehensive Cancer Organization, most recently updated in July 2022. Recurrence was defined as either locoregional recurrence (in colon, rectum, or surrounding lymphatic tissue) or distant metastasis occurring after CRC surgery. Follow-up time was calculated from date of surgery to date of recurrence, date of death, censor date, or loss to follow-up (eg, moving abroad), whichever occurred first. If participants underwent surgery but the surgery date was not available (*n* = 21), we used the CT-scan date as start of follow-up. For explorative analyses, we focused on liver metastases specifically, with follow-up time being calculated from date of surgery until date of liver metastasis, date of death, censor date, or loss to follow-up, whichever occurred first.

Data on OS were retrieved through linkage with the Personal Records Database in November 2024. Events were defined as death from any cause. Follow-up time was calculated from date of surgery to date of death, censor date, or loss to follow-up, whichever occurred first. If the surgery date was not available (*n* = 21), we used the CT-scan date.

For analyses on RFS, events were defined as recurrence or death from any cause. Follow-up time was calculated starting from date of surgery. For explorative analyses focusing on hepatic RFS, events were defined as liver metastasis or death from any cause.

For crude Kaplan-Meier curves, we presented 5-year study endpoints. Here, we considered events occurring within 5 years after surgery, and maximum follow-up time was therefore set at 1826 days.

### Other covariates

Self-reported height (kg) and weight (m) at diagnosis were used to calculate BMI (kg/m^2^). Smoking status at diagnosis (current/former/never) was self-reported using a questionnaire. Moderate-to-vigorous physical activity at diagnosis (minutes/week), defined as a metabolic equivalent (MET) score ≥3,^35^ was assessed using the validated Short Questionnaire to ASsess Health-enhancing physical activity (SQUASH).^36^ Habitual alcohol intake in the month before diagnosis (g of ethanol/day) was assessed with a semi-quantitative food frequency questionnaire (FFQ). Data on pre-existing diabetes and cardiovascular disease (CVD) were collected via the Dutch ColoRectal Audit (DCRA)^37^ supplemented with self-reported data on pre-existing disease and medication use.^38^ Data on treatment, including (neo-)adjuvant chemotherapy and/or radiotherapy (yes/no), cancer stage (I/II/III), and tumor location (colon, defined as caecum to sigmoid colon [including ICD-O-3 C18.0 to C18.9]; or rectum, defined as rectosigmoid junction and rectum [ICD-O-3 C19.9 and C20.9]) were collected via the NCR, medical records, and DCRA.

### Statistical analyses

Characteristics of the total study population and by sex-specific tertiles of liver fat for individuals with colon or rectal cancer are presented with medians (quartile [Q] 1-3) for continuous data, and numbers (percentages) for categorical data.

Restricted cubic splines (RCS) were used to investigate nonlinearity of the associations. Knots were placed at the 10th, 50th, and 90th percentiles, and the graph was truncated at the 1st and 99th percentile. The median of the lowest sex-specific tertile of liver fat (ie, tertile with highest liver radiodensity) was used as the reference. Wald’s statistic was used to assess nonlinearity of the associations, with *P*-values < 0.05 indicating nonlinear associations.^39^

If associations did not appear nonlinear, we used Cox proportional hazards regression with continuous and categorical exposures to calculate hazard ratios (HRs) and 95% confidence intervals (CIs). For continuous analyses, liver fat was evaluated per 20 HU decrement (ie, increase in liver fat). For categorical analyses, the lowest sex-specific tertile for liver fat was used as the reference. Crude models were sex- and age-adjusted. Thereafter, we constructed 2 models in which we included a set of a priori defined covariates based on the literature. The first model included cancer stage and additional treatment received ([neo-]adjuvant chemotherapy and/or radiotherapy).^4^^,^^40^ For the second model, we additionally included alcohol intake, smoking status, and moderate-to-vigorous physical activity,^41-44^ to evaluate the potential impact of lifestyle on risk estimates. For associations of liver fat with RFS and OS, pre-existing diabetes and CVD were also added to the second model.^45-48^ We further constructed models additionally adjusted for BMI, to assess associations independent of adiposity.^49^ All analyses were performed for the overall population and stratified by primary tumor location. *I*^2^-statistics and corresponding *P*-values for heterogeneity between colon and rectal cancer were assessed using the metafor package in R. To address potential reverse causality,^50^^,^^51^ we performed sensitivity analyses in which individuals who developed a recurrence or died within 6 months after surgery were excluded (*n* = 14 and *n* = 18, respectively).

All analyses were performed in R (version 4.3.2). A 95% CI not including 1 or *P*-value < .05 was considered statistically significant.

## Results

Median [Q1-Q3] age of the study population at diagnosis was 67 [61-73] years, 37% were women, and 68% had a tumor located in the colon (vs 32% in the rectum) (Table 1). Individuals with rectal cancer were less often women (30% vs 39%) and more likely to have stage III cancer (48% vs 37%) as compared to individuals with colon cancer (Table 1). Individuals with more liver fat (T3) had a higher median BMI compared to individuals with less liver fat (T1) (27.7 vs 25.2 kg/m^2^ for colon and 27.7 vs 24.9 kg/m^2^ for rectal cancer).

**Table 1.** Baseline characteristics for the overall study population and according to sex-specific tertiles of liver fat for study participants with colon or rectal cancer.

	Colorectal	Colon			Rectal		
		Sex-specific tertiles for liver fat^a^			Sex-specific tertiles for liver fat^a^		
	Total (*n* = 1596)	T1 (*n* = 360)	T2 (*n* = 360)	T3 (*n* = 360)	T1 (*n* = 173)	T2 (*n* = 172)	T3 (*n* = 171)
Liver radiodensity, HU	102 [91-115]	120 [113-130]	103 [98-106]	86 [79-91]	120 [115-128]	102 [98-106]	87 [77-92]
Age, years	67 [61-73]	67 [62-72]	67 [61-73]	68 [63-73]	66 [60-71]	66 [60-73]	65 [61-70]
Sex							
Women	583 (37)	142 (39)	142 (39)	142 (39)	53 (31)	52 (30)	52 (30)
Men	1013 (63)	218 (61)	218 (61)	218 (61)	120 (69)	120 (70)	119 (70)
Body mass index,^b^ kg/m^2^	26.2 [24.0-28.8]	25.2 [23.1-27.6]	25.6 [23.4-28.4]	27.7 [25.1-30.9]	24.9 [22.9-27.0]	26.0 [24.5-28.7]	27.7 [25.4-31.5]
Time spent on moderate-to-vigorous physical activity, min/week^c^	670 [330-1170]	723 [360-1180]	720 [333-1200]	565 [289-1110]	660 [343-1280]	720 [430-1140]	605 [300-1080]
Alcohol intake, g/day^d^	8.0 [1.0-20.0]	8.1 [1.0-17.5]	5.3 [1.0-19.1]	7.4 [0.6-21.9]	9.2 [2.0-17.0]	8.4 [1.1-20.4]	9.5 [1.1-21.9]
Smoking status^e^							
Current	156 (10)	26 (7)	39 (11)	32 (9)	26 (15)	15 (9)	18 (11)
Former	874 (55)	202 (56)	184 (51)	199 (55)	93 (54)	93 (54)	103 (60)
Never	466 (29)	107 (30)	119 (33)	100 (28)	48 (28)	53 (31)	39 (23)
Pre-existing CVD	879 (55)	183 (51)	188 (52)	227 (63)	93 (54)	87 (51)	101 (59)
Pre-existing diabetes	197 (12)	25 (7)	45 (13)	63 (18)	19 (11)	17 (10)	28 (16)
Cancer stage							
I	449 (28)	122 (34)	95 (26)	89 (25)	54 (31)	44 (26)	45 (26)
II	497 (31)	118 (33)	135 (38)	121 (34)	34 (20)	44 (26)	45 (26)
III	650 (41)	120 (33)	130 (36)	150 (42)	85 (50)	84 (50)	81 (47)
Neo-adjuvant treatment,^f^ yes	311 (19)	2 (1)	3 (1)	2 (1)	80 (46)	117 (68)	107 (63)
Adjuvant treatment,^g^ yes	393 (25)	110 (31)	127 (35)	130 (36)	8 (5)	3 (2)	15 (9)
CRC recurrence, yes	247 (15)	35 (10)	43 (12)	55 (15)	41 (23)	43 (25)	30 (18)
Site of CRC recurrence							
Locoregional^h^	65 (26)	8 (23)	14 (33)	11 (20)	13 (32)	14 (33)	5 (17)
Distant^h^	217 (88)	32 (91)	41 (95)	53 (96)	33 (80)	33 (77)	25 (83)
Liver	103 (42)	14 (40)	20 (47)	28 (51)	15 (37)	15 (35)	11 (37)
Lung	92 (37)	8 (23)	19 (44)	14 (24)	15 (37)	21 (49)	15 (50)
Peritoneum	30 (12)	7 (20)	5 (12)	16 (29)	2 (5)	0 (0)	0 (0)
Other	73 (30)	13 (37)	12 (28)	19 (35)	9 (22)	9 (21)	11 (37)
RFS event,^i^ yes	478 (30)	72 (20)	97 (27)	123 (34)	62 (36)	67 (39)	57 (33)
Death, yes	418 (26)	68 (19)	86 (24)	115 (32)	47 (27)	52 (30)	50 (29)

Data are presented as median [Q1-Q3] or number (%). All characteristics were evaluated at time of diagnosis unless indicated otherwise.

Abbreviations: CVD = cardiovascular disease; CRC = colorectal cancer; HU = Hounsfield units; RFS = recurrence-free survival.

a Lowest liver radiodensity value reflects the highest amount of liver fat.

b Data are missing for 43 participants.

c 104 participants.

d 112 participants.

e 100 participants.

f Any type of neo-adjuvant treatment, missing for 15 participants.

g Any type of adjuvant treatment, missing for 16 participants.

h Percentage as part of all recurrences. Percentages do not add up since participants could be diagnosed with multiple recurrences.

i RFS events were defined as any recurrence or death of any cause.

Individuals with colon cancer with the highest amount of liver fat (T3) spent less time on moderate-to-vigorous physical activity, and were more likely to have stage III cancer, pre-existing CVD, or diabetes, compared to individuals with the lowest amount of liver fat (T1) (Table 1). Individuals with rectal cancer in T2 and T3 were more likely to receive neo-adjuvant treatment compared to T1.

During a median follow-up of 6.4 [4.0-7.6] years, a total of 247 (15%) recurrences occurred (12% for colon and 22% for rectal cancer), of which 103 (42%) were liver metastases (47% for colon and 36% for rectal cancer). For RFS, 478 (30%) events occurred (27% for colon and 35% for rectal cancer), of which 247 were recurrences and 231 were deaths. Moreover, during a median follow-up of 8.8 [6.5-10.3] years, 418 (26%) deaths occurred (25% for colon and 29% for rectal cancer).

**Figure 2.**
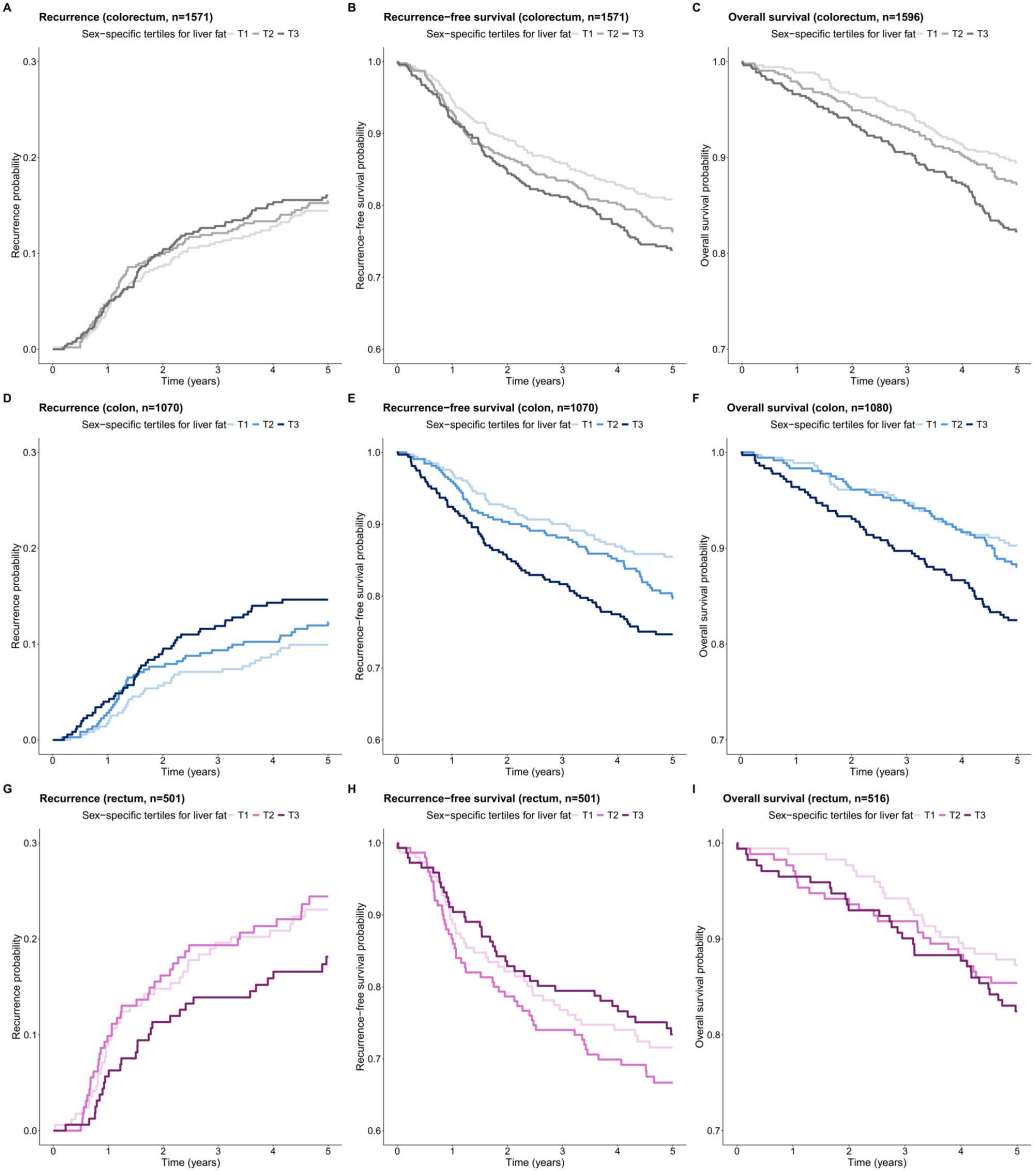
Kaplan-Meier curves showing 5-year recurrence, recurrence-free survival, and overall survival probabilities for individuals with colorectal (total study population; panel A to C), colon (panel D to F), and rectal cancer (panel G to I).

Kaplan-Meier curves, with sex-specific tertiles of liver fat as grouping factor, showed diverging patterns of 5-year recurrence, RFS and OS for colon versus rectal cancer (Figure 2). When evaluating the RCS, none of the associations appeared nonlinear (Figure S1).

More liver fat was associated with increased risk of recurrence (HR_T3vsT1_ = 1.60, 95% CI = 1.02 to 2.50) in individuals with colon cancer. For rectal cancer no association was found (HR_T3vsT1_ = 0.70, 95% CI = 0.42 to 1.18) (*I*^2^ = 82%, *P*_heterogeneity_ = .02) (Table 2). Additionally adjusting for BMI did not markedly change these results (HR_T3vsT1_ = 1.61, 95% CI = 1.01 to 2.57 for colon and HR_T3vsT1_ = 0.73, 95% CI = 0.42 to 1.28 for rectal cancer) (*I*^2^ = 78%, *P*_heterogeneity_ = .03).

**Table 2.** Associations of liver fat with recurrence, recurrence-free survival, and overall survival in participants with stage I-III colorectal (total study population), colon or rectal cancer.

Recurrence	Colorectal		Colon		Rectal	
	*n*/events	HR (95% CI)	*n*/events	HR (95% CI)	*n*/events	HR (95% CI)
Crude model^a^						
Continuous^b^	1571/247	1.04 (0.91 to 1.18)	1070/133	1.18 (0.99 to 1.42)	501/114	0.89 (0.73 to 1.08)
T1	526/77	1.00 (ref)	356/35	1.00 (ref)	171/41	1.00 (ref)
T2	521/83	1.10 (0.81 to 1.50)	356/43	1.24 (0.79 to 1.93)	165/43	1.11 (0.72 to 1.70)
T3	524/87	1.14 (0.84 to 1.55)	358/55	1.62 (1.06 to 2.48)	165/30	0.73 (0.45 to 1.17)
Model 1^c^						
Continuous	1568/246	1.00 (0.87 to 1.14)	1068/132	1.14 (0.95 to 1.37)	500/114	0.86 (0.70 to 1.05)
T1	524/76	1.00 (ref)	355/34	1.00 (ref)	170/41	1.00 (ref)
T2	521/83	1.02 (0.75 to 1.40)	356/43	1.18 (0.75 to 1.85)	165/43	1.09 (0.70 to 1.69)
T3	523/87	1.05 (0.77 to 1.43)	357/55	1.48 (0.96 to 2.27)	165/30	0.70 (0.43 to 1.14)
Model 2^d^						
Continuous	1458/222	1.02 (0.88 to 1.18)	988/122	1.19 (0.97 to 1.44)	470/100	0.86 (0.69 to 1.07)
T1	494/69	1.00 (ref)	333/32	1.00 (ref)	162/36	1.00 (ref)
T2	491/75	1.02 (0.74 to 1.42)	335/39	1.13 (0.71 to 1.81)	154/38	1.11 (0.70 to 1.77)
T3	473/78	1.10 (0.79 to 1.53)	320/51	1.60 (1.02 to 2.50)	154/26	0.70 (0.42 to 1.18)
Recurrence-free survival	Colorectal		Colon		Rectal	
Crude model^a^						
Continuous	1571/478	1.07 (0.97 to 1.18)	1070/292	1.18 (1.04 to 1.34)	501/186	0.93 (0.80 to 1.09)
T1	526/135	1.00 (ref)	356/72	1.00 (ref)	171/62	1.00 (ref)
T2	521/160	1.12 (0.89 to 1.41)	356/97	1.22 (0.90 to 1.66)	165/67	1.05 (0.74 to 1.48)
T3	524/183	1.23 (0.99 to 1.54)	358/123	1.53 (1.14 to 2.05)	165/57	0.84 (0.58 to 1.20)
Model 1^c^						
Continuous	1568/476	1.05 (0.95 to 1.16)	1068/291	1.16 (1.03 to 1.32)	500/185	0.91 (0.77 to 1.06)
T1	524/133	1.00 (ref)	355/71	1.00 (ref)	170/61	1.00 (ref)
T2	521/160	1.09 (0.87 to 1.38)	356/97	1.21 (0.89 to 1.65)	165/67	1.05 (0.74 to 1.50)
T3	523/183	1.20 (0.96 to 1.50)	357/123	1.49 (1.11 to 2.00)	165/57	0.82 (0.56 to 1.18)
Model 2^e^						
Continuous	1458/424	1.04 (0.93 to 1.16)	988/259	1.12 (0.98 to 1.29)	470/165	0.94 (0.79 to 1.12)
T1	494/119	1.00 (ref)	333/65	1.00 (ref)	162/53	1.00 (ref)
T2	491/145	1.11 (0.87 to 1.42)	335/87	1.16 (0.84 to 1.61)	154/60	1.16 (0.79 to 1.70)
T3	473/160	1.19 (0.93 to 1.52)	320/107	1.45 (1.05 to 2.00)	154/52	0.87 (0.59 to 1.30)
Overall survival	Colorectal		Colon		Rectal	
Crude model^a^						
Continuous	1596/418	1.17 (1.05 to 1.30)	1080/269	1.25 (1.10 to 1.42)	516/149	1.05 (0.88 to 1.25)
T1	533/115	1.00 (ref)	360/68	1.00 (ref)	172/47	1.00 (ref)
T2	532/136	1.17 (0.91 to 1.50)	360/86	1.22 (0.87 to 1.66)	171/52	1.09 (0.74 to 1.62)
T3	531/167	1.46 (1.15 to 1.86)	360/115	1.67 (1.24 to 2.25)	171/50	1.08 (0.72 to 1.61)
Model 1^c^						
Continuous	1590/415	1.15 (1.04 to 1.28)	1077/267	1.24 (1.08 to 1.41)	513/148	1.01 (0.85 to 1.21)
T1	531/113	1.00 (ref)	359/67	1.00 (ref)	171/46	1.00 (ref)
T2	530/136	1.13 (0.88 to 1.46)	360/86	1.19 (0.87 to 1.65)	171/52	1.03 (0.69 to 1.54)
T3	529/166	1.40 (1.10 to 1.78)	358/114	1.60 (1.18 to 2.17)	171/50	1.00 (0.67 to 1.50)
Model 2^e^						
Continuous	1470/363	1.16 (1.03 to 1.30)	992/234	1.21 (1.05 to 1.41)	478/129	1.08 (0.89 to 1.31)
T1	497/97	1.00 (ref)	334/59	1.00 (ref)	163/38	1.00 (ref)
T2	497/122	1.21 (0.92 to 1.58)	338/76	1.18 (0.84 to 1.66)	157/46	1.23 (0.80 to 1.91)
T3	476/144	1.49 (1.14 to 1.94)	320/99	1.67 (1.20 to 2.33)	158/45	1.15 (0.74 to 1.80)

a Adjusted for age (years), sex (women/men).

b Continuous models were evaluated per 20 HU decrement, reflecting an increase in liver fat.

c Crude model + adjusted for cancer stage (I/II/III) and additional treatment received ([neo-adjuvant] chemotherapy and/or radiotherapy [yes/no]).

d Model 1 + adjusted for smoking status (current/former/never), alcohol intake (g/day), and moderate-to-vigorous physical activity (min/week).

e Model 1 + adjusted for smoking status (current/former/never), alcohol intake (g/day), moderate-to-vigorous physical activity (min/week), pre-existing diabetes (yes/no), and pre-existing CVD (yes/no).

Likewise, more liver fat was associated with worse RFS in individuals with colon cancer (HR_T3vsT1_ = 1.45, 95% CI = 1.05 to 2.00), whereas no association was found for rectal cancer (HR_T3vsT1_ = 0.87, 95% CI = 0.59 to 1.30) (*I*^2^ = 74%, *P*_heterogeneity_ = .05) (Table 2). Adjusting for BMI did not notably change these findings (HR_T3vsT1_ = 1.44, 95% CI = 1.03 to 2.00 for colon and 0.86, 95% CI = 0.57 to 1.32 for rectal cancer) (*I*^2^ = 72%, *P*_heterogeneity_ = .06).

More liver fat was also associated with worse OS in individuals with colon cancer (HR_T3vsT1_ = 1.67, 95% CI = 1.20 to 2.33), but not rectal cancer (HR_T3vsT1_ = 1.15, 95% CI = 0.74 to 1.80) (*I*^2^ = 43%, *P*_heterogeneity_ = .19) (Table 2). Further adjustment for BMI did not change these results (HR_T3vsT1_ = 1.70, 95% CI = 1.21 to 2.39 for colon and HR_T3vsT1_ = 1.11, 95% CI = 0.70 to 1.77 for rectal cancer) (*I*^2^ = 53%, *P*_heterogeneity_ = .15).

After excluding individuals who developed a recurrence or died within 6 months after surgery, associations were slightly attenuated in terms of risk estimates and statistical significance, but the direction of the associations did not markedly change (Table S2). Moreover, explorative analyses focusing on liver metastases showed patterns similar to the main analyses (colon: HR_T3vsT1_ = 2.12, 95% CI = 1.09 to 4.14 and rectum: HR_T3vsT1_ = 0.75, 95% CI = 0.34 to 1.67) (*I*^2^ = 74%, *P*_heterogeneity_ = .05). The same pattern was observed for hepatic RFS (colon: HR_T3vsT1_ = 1.55, 95% CI = 1.13 to 2.13 and rectum: HR_T3vsT1_ = 0.99, 95% CI = 0.65 to 1.49) (*I*^2^ = 65%, *P*_heterogeneity_ = .09) (Table S3).

## Discussion

In this prospective study among 1596 individuals with stage I-III CRC, more liver fat was associated with increased risk of recurrence, and worse RFS and OS in individuals with colon, but not rectal cancer. After adjustment for BMI, these associations did not markedly change, indicating that observed associations might be independent of BMI.

Among individuals with colon cancer, liver metastases were most common (46% of all recurrences). Liver fat is thought to facilitate liver metastasis by remodeling the liver microenvironment via local lipid accumulation, immunosuppression, and hepatic stellate cell activation.^18^^,^^52^ Also, one could speculate that liver fat may affect chemotherapeutic drug metabolism,^53-55^ potentially resulting in a higher risk for recurrences in individuals with colon cancer. These hypotheses warrant further exploration in future studies.

In line with our findings, a previous study among 604 individuals with stage I-III CRC showed that excessive liver fat, defined as liver-to-spleen-ratio <1.1 measured on non-enhanced CT, was associated with worse hepatic RFS (HR = 7.81, 95% CI = 1.72 to 138.0).^26^ Moreover, a nested case-control study showed an increased risk of metachronous liver metastases among 414 individuals with stage I-III CRC with versus without excessive liver fat at diagnosis (OR = 1.99, 95% CI = 1.19 to 3.30).^8^ It should be noted that the latter study also measured liver fat on non-enhanced CT, as opposed to our use of contrast-enhanced CT. Although contrast-enhancement may result in underestimation of liver fat content,^56^ this will likely be non-differential in our study since we used the same scanning phase for all participants. Recent work demonstrates that liver radiodensity values are suitable and preferred over liver-to-spleen-ratios for assessment of moderate amounts of liver fat on contrast-enhanced CT.^57^ Considering the evidence presented above, excessive liver fat may be considered a risk factor for colon cancer recurrence.

We also observed that liver fat was associated with worse OS in individuals with colon, but not rectal, cancer. Another study found that a previous ICD-9 or ICD-10 diagnosis of non-alcoholic fatty liver disease (NAFLD) was associated with increased risk for OS among 3262 individuals with stage I-III CRC (HR = 1.64, 95% CI = 1.06 to 2.54).^7^ There is some evidence suggesting that liver fat is also associated with increased risk for CRC-specific mortality in individuals diagnosed with non-metastatic CRC.^7,25^ Together with the observed relationships between liver fat and cancer recurrence and consequently survival in individuals with colon cancer, and the absence of associations in individuals with rectal cancer, our results may indicate that liver fat is linked to survival via colon cancer-specific pathways. On the other hand, it cannot be ruled out that cardiometabolic events occurring during follow-up mediated relationships between liver fat and survival in individuals with colon cancer,^48^ even though we adjusted for pre-existing cardiometabolic conditions.

No clear associations between liver fat and clinical outcomes were observed among individuals with rectal cancer. The only study conducted so far among 241 individuals with stage II-III rectal cancer neither showed associations between liver fat (liver-to-spleen-ratio <1.1 on non-enhanced CT) and risk of metachronous liver metastases (HR = 1.25, 95% CI = 0.55 to 2.85) nor OS (HR = 0.66, 95% CI = 0.35 to 1.22),^24^ which may also be explained by the relatively modest sample size. Rectal cancer commonly disseminates to both liver and lungs, the latter via rectal veins and systemic circulation, while colon cancer predominantly metastasizes to the liver via the portal vein.^4^^,^^5^^,^^30^^,^^58^^,^^59^ We observed a relatively lower number of liver metastases compared to lung metastases within our rectal cancer population (respectively, 36% and 45% of all recurrences in rectal cancer), which may explain why associations with liver fat are not pronounced. Moreover, tumors originating in the rectum have different molecular characteristics as compared to colon tumors^60^ and cancer dissemination patterns are tumor-specific according to the “seed and soil” hypothesis.^5^^,^^61^ Hence, it could be hypothesized that colon cancer cells may “seed” better in a fatty liver as compared to rectal cancer cells, which warrants further confirmation in future research.

Potential limitations of our study should be considered. Due to the observational design, causality cannot be demonstrated and we cannot fully rule out possible reverse causation. Excluding individuals with a recurrence or death within 6 months after surgery did only slightly impact our findings. Although possible reduced statistical power due to a lower number of events cannot be excluded, these findings may imply that liver fat could mask liver metastases on CT-scans. These findings further emphasize the potential importance of liver fat in colon cancer diagnosis and prognosis. Moreover, we did not define excessive liver fat based on clinically relevant cut-off values, but assessed liver fat as a continuous measure. Nevertheless, we were able to investigate potential nonlinearity in our associations due to this approach, which could ultimately help to define clinically relevant cut-off values for liver fat on contrast-enhanced CT. We were not able to investigate liver fibrosis,^62^ since we did not have sufficient clinical data to calculate liver fibrosis indices such as FIB-4,^63^ and future research is therefore warranted to evaluate the full spectrum of liver disease in relation to CRC prognosis. Furthermore, we were not able to consider resection status in our analyses, since data collection on resection status by the DCRA was initiated 5 years after the start of our study and data were therefore only available for 804 (50%) participants. However, in this subset, only 17 (2%) patients did have a R1 resection status indicating residual disease, which is in line with population-based data.^64^ Lastly, we were not able to collect data on cause of death in our study due to privacy regulations; hence, we were not able to confirm hypotheses related to cancer-related survival.

This study also has several important strengths. The prospective design and relatively large sample size enabled us to investigate associations of liver fat with recurrence, RFS and OS for colon and rectal cancer separately. Moreover, we had comprehensive follow-up data on CRC recurrence and survival. Since the majority of CRC recurrences occur within 2-3 years,^4^^,^^65^ our median follow-up time of 6.4 years was sufficient to capture most recurrences. Furthermore, given the availability of both lifestyle and clinical data, we were able to adjust for relevant confounders.

In conclusion, we found that liver fat was associated with increased risks for recurrence and liver metastases, and worse (recurrence-free) survival in individuals with colon, but not rectal cancer. These findings highlight the potential role of liver fat in colon cancer prognosis.

## Supplementary Material

djaf324_Supplementary_Data

## Data Availability

Since the data consist of identifying cohort information, some access restrictions apply and therefore individual patient data cannot be made publicly available. Data will be shared with permission from the steering committee of the COLON study. Requests for data can be sent to Dr Dieuwertje Kok, Division of Human Nutrition and Health, Wageningen University & Research, The Netherlands (dieuwertje.kok@wur.nl).
