# Supplementary material for: Liver fat and clinical outcomes in individuals with stage I-III colon or rectal cancer
Source: J Natl Cancer Inst. 2025 Nov 10;118(3):466–75. doi: 10.1093/jnci/djaf324 (PMC13016977; doi:10.1093/jnci/djaf324)
Supplement: djaf324_Supplementary_Data [file djaf324_supplementary_data.zip › Ophoff_Supplementary data_Revised.pdf]

SUPPLEMENTARY MATERIAL

Table S1: Characteristics for participants with a portal phase CT-scan and participants without a portal phase CT-scan.

	Current study population with portal phase CT-scan available (n=1596)	No portal phase CT-scan available (n=267)
Age, years	67<br>[61 to 73]	66<br>[61 to 71]
Sex<br>Women<br>Men	 583 (37)<br>1013 (63)	 92 (34)<br>175 (66)
BMI[a], kg/m^2	26.2<br>[24.0 to 28.8]	26.1<br>[23.9 to 28.7]
Time spent on moderate-to-vigorous physical activity[b], min/week	670<br>[330 to 1170]	710<br>[370 to 1110]
Alcohol intake[c], g/day	8.0<br>[1.0 to 20.0]	9.1<br>[1.1 to 23.3]
Smoking status[d]<br>Current<br>Former<br>Never	 156 (10)<br>874 (55)<br>466 (29)	 18 (7)<br>147 (55)<br>75 (28)
Pre-existing CVD, yes	879 (55)	150 (56)
Pre-existing diabetes, yes	197 (12)	29 (11)
Tumour location<br>Colon<br>Rectum	 1080 (68)<br>516 (32)	 178 (67)<br>89 (33)
Cancer stage<br>I<br>II<br>III	 449 (28)<br>497 (31)<br>650 (41)	 95 (36)<br>63 (24)<br>109 (41)
Neo-adjuvant treatment[e], yes	311 (19)	56 (21)
Adjuvant treatment[f], yes	393 (25)	64 (24)
CRC recurrence, yes	247 (15)	40 (15)
Site of CRC recurrence[g]<br>Locoregional recurrence<br>Distant metastasis	 65 (26)<br>217 (88)	 9 (23)<br>38 (95)
RFS event[h], yes	478 (30)	80 (30)
Death, yes	418 (26)	65 (24)

Data are presented as median [Q1-Q3] or numbers (%). All characteristics were evaluated at time of diagnosis unless indicated otherwise.
[a] Data are missing for respectively 43 and 17 participants; [b] 104 and 27 participants; [c] 112 and 30 participants; [d] 100 and 27 participants; [e] Any type of neo-adjuvant treatment, missing for 15 and 5 participants; [f] Any type of adjuvant treatment, missing for 16 and 5 participants; [g] Percentage as part of all recurrences. Percentages do not add up since participants could develop multiple recurrences at once; [h] RFS events were defined as recurrence or death of any cause.

Table S2: Sensitivity analyses for liver fat in association with recurrence, recurrence-free survival and overall survival **excluding patients who developed a recurrence or died within 6 months after surgery**.

Recurrence	Colorectal		Colon		Rectal	
	n/events	HR (95%CI)	n/events	HR (95%CI)	n/events	HR (95%CI)
Crude model[a]						
Continuous[b]	1557/233	1.03 (0.89 to 1.18)	1061/124	1.17 (0.97 to 1.41)	496/109	0.89 (0.73 to 1.09)
T1	522/73	1.00 (ref)	355/34	1.00 (ref)	168/38	1.00 (ref)
T2	517/79	1.11 (0.80 to 1.52)	353/40	1.19 (0.75 to 1.88)	164/42	1.17 (0.75 to 1.81)
T3	518/81	1.12 (0.82 to 1.54)	353/50	1.52 (0.98 to 2.35)	164/29	0.76 (0.47 to 1.23)
Model 1[c]						
Continuous	1554/232	0.99 (0.86 to 1.14)	1059/123	1.14 (0.94 to 1.37)	495/109	0.86 (0.70 to 1.05)
T1	520/72	1.00 (ref)	354/33	1.00 (ref)	167/38	1.00 (ref)
T2	517/79	1.03 (0.74 to 1.42)	353/40	1.13 (0.71 to 1.79)	164/42	1.15 (0.74 to 1.81)
T3	517/81	1.03 (0.75 to 1.42)	352/50	1.40 (0.90 to 2.18)	164/29	0.74 (0.45 to 1.21)
Model 2[d]						
Continuous	1446/210	1.01 (0.87 to 1.18)	981/115	1.18 (0.96 to 1.44)	465/95	0.86 (0.69 to 1.08)
T1	490/65	1.00 (ref)	332/31	1.00 (ref)	159/33	1.00 (ref)
T2	488/72	1.04 (0.74 to 1.46)	333/37	1.11 (0.69 to 1.80)	153/37	1.19 (0.74 to 1.93)
T3	468/73	1.08 (0.77 to 1.52)	316/47	1.53 (0.97 to 2.41)	153/25	0.74 (0.43 to 1.25)
Recurrence-free survival						
Crude model[a]						
Continuous	1539/446	1.04 (0.94 to 1.16)	1051/273	1.16 (1.02 to 1.32)	488/173	0.90 (0.77 to 1.06)
T1	519/128	1.00 (ref)	353/69	1.00 (ref)	167/58	1.00 (ref)
T2	512/151	1.11 (0.88 to 1.41)	351/92	1.21 (0.88 to 1.65)	162/64	1.06 (0.74 to 1.52)
T3	508/167	1.18 (0.93 to 1.49)	347/112	1.45 (1.07 to 1.96)	159/51	0.79 (0.54 to 1.16)
Model 1[c]						
Continuous	1536/444	1.03 (0.93 to 1.14)	1049/272	1.15 (1.01 to 1.31)	487/172	0.88 (0.74 to 1.04)
T1	517/126	1.00 (ref)	352/68	1.00 (ref)	166/57	1.00 (ref)
T2	512/151	1.09 (0.86 to 1.38)	351/92	1.20 (0.87 to 1.64)	162/64	1.08 (0.75 to 1.56)
T3	507/167	1.15 (0.91 to 1.45)	346/112	1.42 (1.05 to 1.93)	159/51	0.78 (0.53 to 1.15)
Model 2[e]						
Continuous	1431/397	1.01 (0.91 to 1.13)	974/245	1.10 (0.95 to 1.27)	457/152	0.90 (0.76 to 1.08)
T1	488/113	1.00 (ref)	331/63	1.00 (ref)	158/49	1.00 (ref)
T2	483/137	1.11 (0.86 to 1.42)	331/83	1.14 (0.81 to 1.58)	151/57	1.21 (0.82 to 1.80)
T3	460/147	1.14 (0.88 to 1.46)	312/99	1.36 (0.98 to 1.89)	148/46	0.83 (0.54 to 1.25)
Overall survival						
Crude model[a]						
Continuous	1578/400	1.15 (1.03 to 1.28)	1070/259	1.24 (1.08 to 1.41)	508/141	1.01 (0.85 to 1.21)
T1	530/112	1.00 (ref)	358/66	1.00 (ref)	172/46	1.00 (ref)
T2	527/131	1.16 (0.90 to 1.49)	358/84	1.21 (0.88 to 1.67)	170/50	1.07 (0.72 to 1.61)
T3	521/157	1.41 (1.11 to 1.80)	354/109	1.63 (1.20 to 2.22)	166/45	0.99 (0.66 to 1.50)
Model 1[c]						

Continuous	1572/397	1.13 (1.02 to 1.26)	1067/257	1.22 (1.07 to 1.40)	505/140	0.97 (0.80 to 1.16)
T1	528/110	1.00 (ref)	357/65	1.00 (ref)	171/45	1.00 (ref)
T2	525/131	1.12 (0.87 to 1.45)	358/84	1.20 (0.87 to 1.67)	169/50	1.00 (0.66 to 1.50)
T3	519/156	1.36 (1.06 to 1.74)	352/108	1.57 (1.16 to 2.15)	165/45	0.90 (0.59 to 1.38)
Model 2[e]						
Continuous	1455/348	1.14 (1.01 to 1.28)	985/227	1.20 (1.03 to 1.39)	470/121	1.04 (0.85 to 1.27)
T1	495/95	1.00 (ref)	333/58	1.00 (ref)	163/37	1.00 (ref)
T2	492/117	1.18 (0.90 to 1.56)	336/74	1.16 (0.82 to 1.64)	155/44	1.23 (0.78 to 1.93)
T3	468/136	1.43 (1.10 to 1.88)	316/95	1.63 (1.16 to 2.28)	152/40	1.06 (0.67 to 1.68)

[a] Adjusted for age (years), sex (women/men)
[b] Continuous models were evaluated per 20 HU decrement, reflecting an increase in liver fat
[c] Crude model + adjusted for cancer stage (I/II/III) and additional treatment received ((neo-adjuvant) chemotherapy and/or radiotherapy, yes/no)
[d] Model 1 + adjusted for smoking status (current/former/never), alcohol intake (g/day) and moderate-to-vigorous physical activity (min/week)
[e] Model 1 + adjusted for smoking status (current/former/never), alcohol intake (g/day), moderate-to-vigorous physical activity (min/week), pre-existing diabetes (yes/no), and pre-existing CVD (yes/no)

Table S3: Associations of liver fat with **liver metastases and hepatic recurrence-free survival** in participants with stage I-III colorectal (total study population), colon or rectal cancer.

Liver metastases	Colorectal		Colon		Rectal	
	n/events	HR (95%CI)	n/events	HR (95%CI)	n/events	HR (95%CI)
Crude model[a]						
Continuous[b]	1573/103	1.10 (0.89 to 1.35)	1072/62	1.21 (0.93 to 1.57)	501/41	0.96 (0.70 to 1.34)
T1	526/29	1.00 (ref)	356/14	1.00 (ref)	171/15	1.00 (ref)
T2	523/34	1.20 (0.73 to 1.97)	358/20	1.42 (0.72 to 2.82)	165/15	1.08 (0.53 to 2.22)
T3	524/40	1.42 (0.88 to 2.29)	358/28	2.05 (1.08 to 3.89)	165/11	0.78 (0.36 to 1.71)
Model 1[c]						
Continuous	1570/103	1.06 (0.86 to 1.30)	1070/62	1.14 (0.87 to 1.49)	500/41	0.94 (0.68 to 1.32)
T1	524/29	1.00 (ref)	355/14	1.00 (ref)	170/15	1.00 (ref)
T2	523/34	1.16 (0.70 to 1.90)	358/20	1.37 (0.69 to 2.71)	165/15	1.09 (0.52 to 2.28)
T3	523/40	1.31 (0.82 to 2.15)	357/28	1.81 (0.95 to 3.45)	165/11	0.79 (0.35 to 1.75)
Model 2[d]						
Continuous	1460/97	1.08 (0.87 to 1.35)	990/57	1.21 (0.91 to 1.62)	470/40	0.93 (0.65 to 1.31)
T1	494/28	1.00 (ref)	333/13	1.00 (ref)	162/15	1.00 (ref)
T2	493/31	1.08 (0.65 to 1.82)	337/17	1.25 (0.61 to 2.58)	154/14	0.92 (0.44 to 1.95)
T3	473/38	1.40 (0.86 to 2.30)	320/27	2.12 (1.09 to 4.14)	154/11	0.75 (0.34 to 1.67)
Hepatic recurrence-free survival						
Crude model[a]						
Continuous	1573/445	1.08 (0.97 to 1.19)	1072/280	1.16 (1.02 to 1.32)	501/165	0.96 (0.81 to 1.14)
T1	526/125	1.00 (ref)	356/70	1.00 (ref)	171/55	1.00 (ref)
T2	523/147	1.10 (0.86 to 1.39)	358/91	1.16 (0.85 to 1.58)	165/58	0.99 (0.68 to 1.44)
T3	524/173	1.26 (1.00 to 1.58)	358/119	1.51 (1.12 to 2.03)	165/52	0.87 (0.59 to 1.27)
Model 1[c]						
Continuous	1570/443	1.06 (0.96 to 1.18)	1070/279	1.15 (1.01 to 1.31)	500/164	0.94 (0.79 to 1.11)
T1	524/123	1.00 (ref)	355/69	1.00 (ref)	170/54	1.00 (ref)
T2	523/147	1.08 (0.85 to 1.37)	358/91	1.16 (0.84 to 1.58)	165/58	0.99 (0.68 to 1.45)
T3	523/173	1.23 (0.98 to 1.56)	357/119	1.48 (1.10 to 1.99)	165/52	0.85 (0.57 to 1.26)
Model 2[e]						
Continuous	1460/394	1.09 (0.98 to 1.22)	990/248	1.15 (1.00 to 1.32)	470/146	1.02 (0.85 to 1.22)
T1	494/109	1.00 (ref)	333/63	1.00 (ref)	162/46	1.00 (ref)
T2	493/133	1.12 (0.87 to 1.45)	337/81	1.12 (0.81 to 1.57)	154/52	1.12 (0.75 to 1.69)
T3	473/152	1.33 (1.04 to 1.71)	320/104	1.55 (1.13 to 2.13)	154/48	0.99 (0.65 to 1.49)

[a] Adjusted for age (years), sex (women/men)
[b] Continuous models were evaluated per 20 HU decrement, reflecting an increase in liver fat
[c] Crude model + adjusted for cancer stage (I/II/III) and additional treatment received ((neo-adjuvant) chemotherapy and/or radiotherapy, yes/no)
[d] Model 1 + adjusted for smoking status (current/former/never), alcohol intake (g/day) and moderate-to-vigorous physical activity (min/week)
[e] Model 1 + adjusted for smoking status (current/former/never), alcohol intake (g/day), moderate-to-vigorous physical activity (min/week), pre-existing diabetes (yes/no), and pre-existing CVD (yes/no)

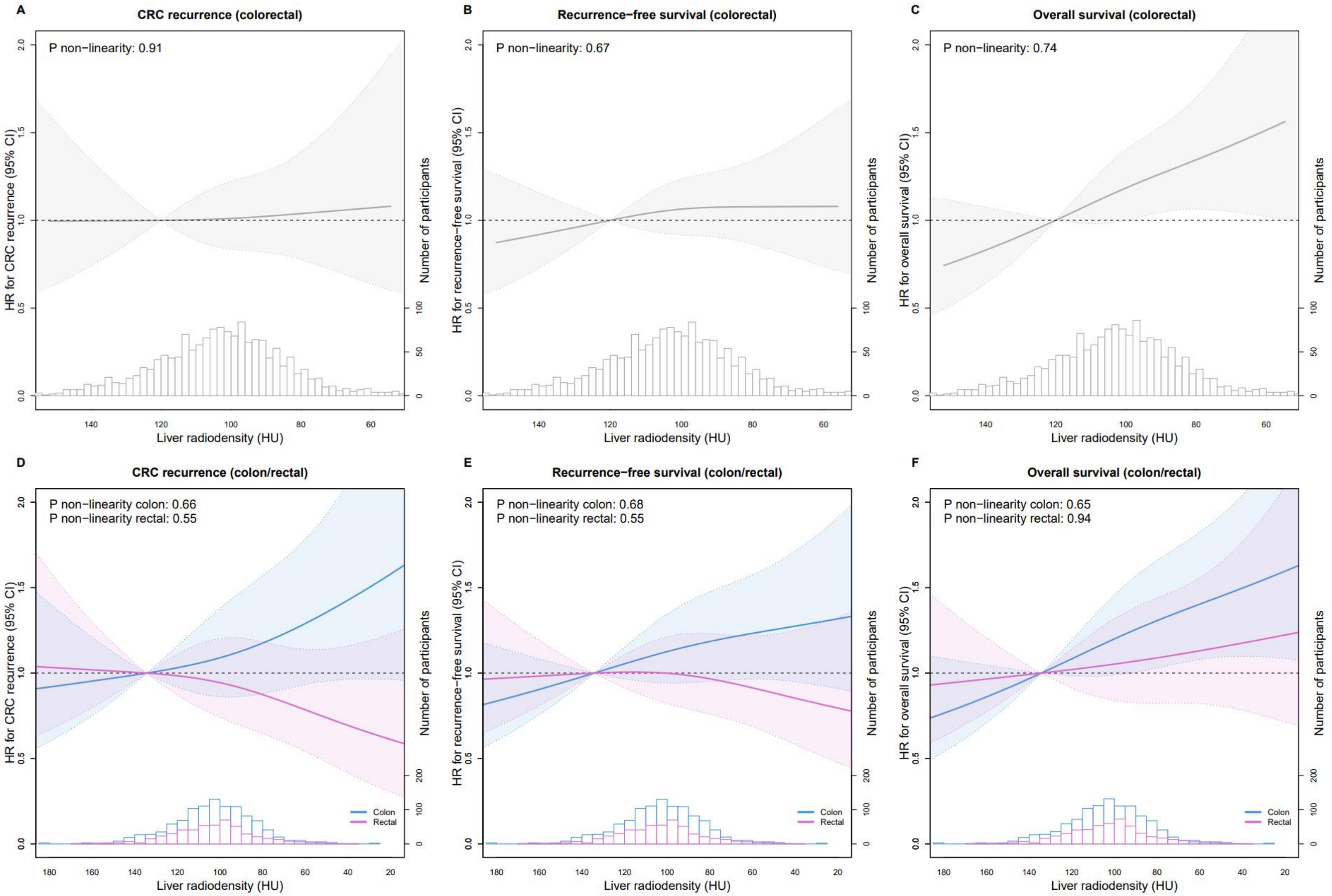

Figure S1: Restricted cubic splines showing the shape of the associations of liver fat with CRC recurrence, recurrence-free survival, and overall survival for the total population (panel A to C) and colon versus rectal cancer (panel D to F). The solid line represents the HR, the dashed lines represent the 95%CIs. Models were adjusted for age, sex, cancer stage (I/II/III), additional treatment received ((neo-adjuvant) chemotherapy and/or radiotherapy, yes/no), smoking status (current/former/never), alcohol consumption (g/day), and moderate-to-vigorous physical activity (min/week). Models for recurrence-free survival and overall survival were additionally adjusted for pre-existing diabetes (yes/no) and pre-existing cardiovascular disease (yes/no).
